# The effect of vitamin D supplementation on glycemic status and C-reactive protein levels in type 2 diabetic patients with ischemic heart disease: A protocol for systematic review and meta-analysis

**DOI:** 10.1097/MD.0000000000032254

**Published:** 2022-12-09

**Authors:** Xuxia Chai, Yonghe Jin, Yongmei Wei, Rong Yang

**Affiliations:** a Department of Clinical Laboratory, Zhangye People’s Hospital Affiliated to Hexi University, Gansu, China.

**Keywords:** diabetes mellitus, ischemic heart diseases, meta-analysis, vitamin D

## Abstract

Vitamin D might be beneficial in diabetic patients with ischemic heart disease through its favorable effect on metabolic profiles and biomarkers of inflammation and oxidative stress. We performed a protocol for systematic review and meta-analysis to assess whether vitamin D supplementation could improve glucose control and inflammation in type 2 diabetic patients with ischemic heart disease.

**Methods:**

The proposed systematic review and meta-analysis will conform to the Preferred Reporting Items for Systematic Review and Meta-analysis Protocols. Seven electronic databases including Web of Science, Embase, PubMed, Wanfang Data, Scopus, Science Direct, Cochrane Library were searched in October 2022 by 2 independent reviewers. The risk of bias assessment of the included studies was assessed using the tool recommended in the Cochrane Handbook for Systematic Reviews of Interventions (version 5.1.0). Data analysis was performed with Review Manager Software (RevMan Version 5.4, The Cochrane Collaboration, Copenhagen, Denmark).

**Results:**

This study will provide a high-quality synthesis to assess the effectiveness and safety of vitamin D supplementation on type 2 diabetic patients with ischemic heart disease.

**Conclusion:**

This systematic review may lead to several recommendations, for both patients and researchers, as which is the best therapy for type 2 diabetic patients with ischemic heart disease and how future studies need to be designed, considering what is available now and what is the reality of the patient.

## 1. Introduction

Type 2 diabetes mellitus (T2DM) and ischemic heart disease (IHD) are comorbidities and the major risk factors of mortality.^[1,2]^ In patients with T2DM, IHD is more likely to be a complex disease characterized by small, diffuse, calcified, multivessel disease and often requires coronary revascularization in addition to optimal medical therapy to control angina.^[3,4]^ It is reported that IHD is responsible for more than 80% of death in people with T2DM.^[5]^ Cognitive impairment, depression, and functional disability are involved in the course history of the disease. Insulin resistance and hyperinsulinemia in people with T2DM cause metabolic disorders; leading to decreased nitric oxide synthesis in blood vessel walls.^[6,7]^ Furthermore, inflammation and oxidative stress play important roles in the development of T2DM and IHD, and the close relationship between increased inflammation and oxidative stress in both diseases is now well defined.^[8,9]^

Some studies have reported that circulating concentrations of vitamin D were low in subjects with T2DM and IHD.^[10,11]^ For instance, a study in patients with T2DM who had a history of IHD found a strong inverse relation between low vitamin D concentrations and prevalent coronary or peripheral cardiovascular disease.^[12]^ The beneficial effects of vitamin D administration on indexes of insulin production and resistance, lipid profiles, and biomarkers of inflammation and oxidative stress may be mediated through its impact on activating insulin receptor expression, the downregulation of cytokine generation, and lower production of parathyroid hormone.^[13]^ High-sensitivity C-reactive protein (hs-CRP) as a biomarker of inflammation has inverse relation with vitamin D that affects parathyroid hormone levels and may decrease hs-CRP concentrations.

Given the glucose-lowering, antiinflammatory, and antioxidant effects of vitamin D, we hypothesized that vitamin D supplementation might be beneficial in diabetic patients with IHD. Therefore, we performed a protocol for systematic review and meta-analysis to evaluate the effects of vitamin D supplementation on glucose control and inflammation status evaluated by hs-CRP levels in T2DM patients with IHD.

## 2. Methods

### 2.1. Protocol registration

The proposed systematic review and meta-analysis will conform to the Preferred Reporting Items for Systematic Review and Meta-analysis (PRISMA) protocols.^[14]^ This protocol is registered with the International Prospective Register of Systematic Reviews (PROSPERO), registration number (CRD42022366624). Ethics application was not required as this study is based on published trials.

### 2.2. Selection of studies

Seven electronic databases including Web of Science, Embase, PubMed, Wanfang Data, Scopus, Science Direct, Cochrane Library were searched in October 2022 by 2 independent reviewers. The established search strategy for PubMed was displayed in Table 1. The reference lists of the included studies were also checked for additional studies that were not identified with the database search. There was no restriction in the dates of publication or language in the search. The key terms used for the search are “vitamin D,” “type 2 diabetes mellitus” and “ischemic heart disease.”

**Table 1:** Search strategy in PubMed.

#1 ischemic heart disease [Title/Abstract]
#2 coronary heart disease [Title/Abstract]
#3 coronary atherosclerotic heart disease [Title/Abstract]
#4 myocardial ischemia [Title/Abstract]
#5 #1 OR #2 OR #3 OR #4
#6 type 2 diabetes mellitus [Title/Abstract]
#7 pathoglycemia [Title/Abstract]
#8 hyperglycemia [Title/Abstract]
#9 insulin resistance [Title/Abstract]
#10 #6 OR #7 OR #8 OR #9
#11 vitamin D [Title/Abstract s]
#12 25(OH)Vit D [Title/Abstract]
#13 1,25(OH)2D [Title/Abstract]
#14 #11 OR #12 OR #13
#15 #5 AND #10 AND #14

### 2.3. Inclusion and exclusion criteria

Study included in this systematic review and meta-analysis had to meet all of the following inclusion criteria in the PICOS order:

1. Participants: T2DM patients with IHD disorder.
2. Intervention: patients received a single-dose administration of vitamin D.
3. Comparator: patients received a matching placebo.
4. Outcomes: glycosylated hemoglobin, fasting blood sugar, hsCRP, 25(OH)Vit D levels and adverse effects.
5. Study design: randomized controlled trials.

The exclusion criteria were as follows:

1. Studies which did not assessed the above outcomes.
2. Lack of a control group.
3. Studies with the following types: case reports, comments or letters, biochemical trials, protocols, conference abstracts, reviews, and retrospective studies or prospective non-randomized studies.

### 2.4. Study selection

**Figure 1.**
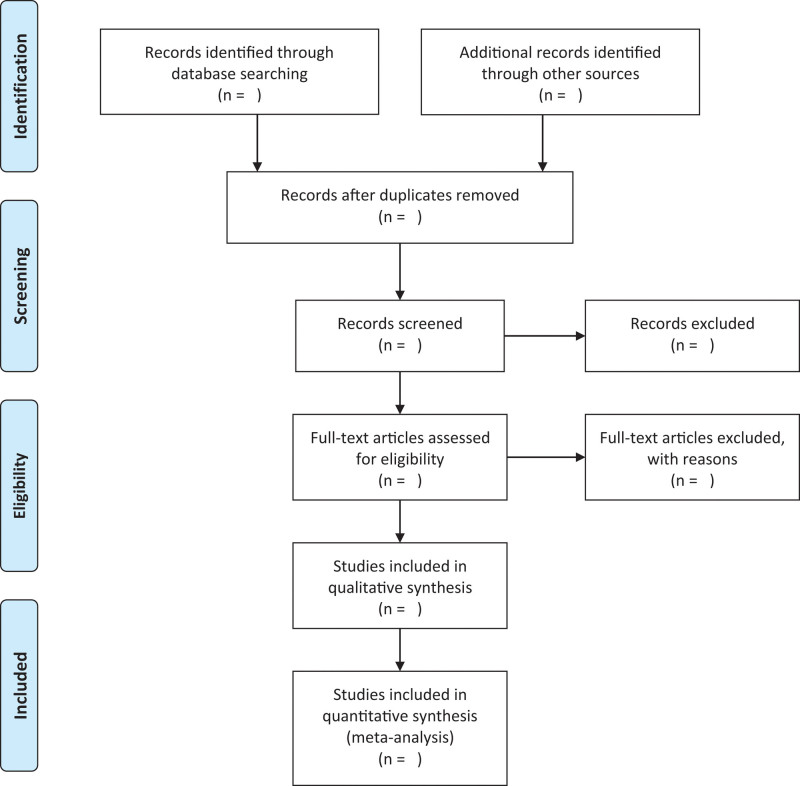
PRISMA flow diagram for study selection. PRISMA = Preferred Reporting Items for Systematic Review and Meta-analysis.

Articles were exported to EndNote, and duplicates removed. Two independent authors screened the titles and abstracts of potentially relevant studies to determine their eligibility based on the criteria. Disagreements were resolved through a discussion with a third review author. The selection process of eligible papers is shown in a Preferred Reporting Items for Systematic Review and Meta-analysis (PRISMA) flow diagram (Fig. 1).

### 2.5. Data extraction

Data were extracted by reviewing each study for population, mean age, gender, follow-up duration, study design, publishing date, intervention methods, and outcomes assessment. The 2 reviewers created a study-specific speadsheet in Excel (Microsoft Corp, USA) for data collection. Any disagreements between the 2 reviewers were discussed and, if necessary, the third author was referred to for arbitration. If the data were missing or could not be extracted directly, authors were contacted by email. Otherwise, we calculated them with the guideline of Cochrane Handbook for Systematic Reviews of Interventions 5.1.0.

### 2.6. Quality assessment

The risk of bias assessment of the included studies was performed by 2 authors independently using the tool recommended in the Cochrane Handbook for Systematic Reviews of Interventions (version 5.1.0).^[15]^ This tool included 7 aspects which were sequence generation (selection bias), allocation sequence concealment (selection bias), blinding of participants and personnel (performance bias), blinding of outcome assessment (detection bias), incomplete outcome data (attrition bias), selective outcome reporting (reporting bias) and other bias (baseline balance and fund). Additionally, each of the aspects was ranked low risk of bias, high risk of bias, and unclear risk of bias. The evidence grade was assessed using the guidelines of the GRADE (Grading of Recommendations, Assessment, Development, and Evaluation) working group^[16]^ including the following items: risk of bias, inconsistency, indirectness, imprecision and publication bias.

The recommendation level of evidence was classified into the following categories:

1. high, which means that further research is unlikely to change confidence in the effect estimate;
2. moderate, which means that further research is likely to significantly change confidence in the effect estimate but may change the estimate;
3. low, which means that further research is likely to significantly change confidence in the effect estimate and to change the estimate; and
4. very low, which means that any effect estimate is uncertain.

GRADE pro Version 3.6 software is used for the evidence synthesis.

### 2.7. Statistical analysis

Data analysis was performed with Review Manager Software (RevMan Version 5.4, The Cochrane Collaboration, Copenhagen, Denmark). The risk ratio and 95% confidence intervals (CIs) were collected for enumeration data, while the mean difference or standardized mean difference and 95% confidence intervals (CIs) were used to calculate continuous outcome data. The heterogeneity of the data was tested by calculating *I*^*2*^ statistics. The study was not considered to have a large heterogeneity when the *I*^*2*^ value was <50%. When the *I*^*2*^ value exceeded 50%, there was significant statistical heterogeneity among the trials. When there is homogeneity in the merged outcome results across sufficient studies, a meta-analysis will be conducted. Otherwise, we performed a subgroup analysis to explore the causes of the heterogeneity.

## 3. Discussion

To the best of our knowledge, this is the first meta-analysis to assess the effects of vitamin D supplementation on glucose control and inflammation status evaluated by hs-CRP levels in T2DM patients with IHD. Focus on the use of vitamin D in non-communicable disorders including diabetes, hypertension and cardiovascular diseases originated from several observational studies showing the detrimental impact of low vitamin D on these disorders.^[17,18]^ Recently, a meta-analysis revealed an association between high levels of 25(OH)Vit D and a 43% decline in cardiovascular disease, T2DM and metabolic syndrome.^[19]^ However, previous studies were criticized by small sample size and methodological errors. This meta-analysis of randomized controlled trials may provide high level evidence for clinicians in order to make decisions when dealing with T2DM patients with IHD.

## Author contributions

**Conceptualization**: Yonghe Jin.

**Finish manuscript**: Xuxia Chai.

**Formal analysis**: Yongmei Wei.

**Software**: Yongmei Wei.

**Validation**: Yongmei Wei.

**Writing – original draft**: Xuxia Chai.

**Writing – review & editing:** Rong Yang.
